# Outcomes following renal transplantation in older people: a retrospective cohort study

**DOI:** 10.1186/1471-2318-13-79

**Published:** 2013-07-24

**Authors:** Niall J Dempster, Carlo DL Ceresa, Emma Aitken, David Kingsmore

**Affiliations:** 1 Department of Renal Surgery and Transplantation, Western Infirmary, Glasgow G11 6NY, UK

**Keywords:** Renal, Kidney, Transplantation, Outcomes, Older patients

## Abstract

**Background:**

The mean age of renal transplant recipients is rising, with age no longer considered a contraindication. Outcomes in older patients have not, however, been fully defined. The aim of our study is to evaluate outcomes in older people following renal transplantation at a Scottish regional transplant unit.

**Methods:**

All renal transplants from January 2001 to December 2010 were analysed (n = 762). Outcomes following renal transplantation in people over 65 years old were compared to those in younger patients. Outcome measures were: delayed graft function (DGF), primary non-function (PNF), biopsy proven acute rejection (BPAR), serum creatinine at 1 year and graft and recipient survival. Lengths of initial hospital stay and re-admission rates were also assessed. Student’s T-Test was used to analyse continuous variables, Pearson’s Chi-Squared test for categorical variables and the Kaplan-Meier estimator for survival analysis.

**Results:**

Older recipients received proportionately more kidneys from older donors (27.1% vs. 6.3%; p  <  0.001). Such kidneys were more likely to have DGF (40.7% vs. 16.9%; p < 0.001). Graft loss at 1 year was higher in kidneys from older donors (15.3% vs. 7.6%; p = 0.04). There was no significant difference in patient survival at 1 year based on the age of the donor kidney. Recipient age did not affect DGF (16.9% vs. 18.5%; p = 0.77) or graft loss at 1 year (11.9% vs. 7.8%; p = 0.28). Older recipients were, however, more likely to die in the first year post transplant (6.8% vs. 2.1%; p = 0.03). BPAR was less common in older patients (6.8% vs. 22%; p < 0.01). Older recipients were more likely to be readmitted to hospital (31.8% vs. 10.9%; p < 0.001).

**Conclusions:**

Older patients experience good outcomes following renal transplantation and donor or recipient age alone should not preclude this treatment. An awareness of this in clinicians managing older patients is important since the prevalence of End Stage Renal Disease is increasing in this age group.

## Background

The incidence and prevalence of older patients with End Stage Renal Disease (ESRD) is increasing, with a median patient age of 64.9 years at time of diagnosis in the United Kingdom [1]. Half of patients with ESRD in the United States are over 65 and one third over 70 years old when first diagnosed [2]. An awareness of the relative benefits and risks of treatment options for older patients with ESRD is therefore increasingly important to the practicing gerontologist.

The principal modalities of renal replacement therapy (RRT) for older patients with ESRD are either dialysis (peritoneal or haemodialysis) or renal transplantation [3]. The mean age of renal transplant recipients is rising, with advanced age no longer considered a contraindication to transplantation. Survival, quality of life and economic advantages have been demonstrated after renal transplantation in older patients, including those with co-morbidities, compared to treatment with dialysis [4-6].

Nevertheless, access to renal transplantation continues to be limited for older people with ESRD. Improvements in access have been at best modest in several European countries including Scotland [7]. Reasons for this may include organ allocation policy, lack of awareness or ageism [8,9], with a Canadian study finding that 26.5% of patients aged 65 or older without contraindications to transplantation were not referred for assessment [10].

Greater definition of outcomes following renal transplantation in patients at the extremes of age would be beneficial. The primary objective of this study was to evaluate graft and patient outcomes following renal transplantation in older donors and recipients.

## Methods

### Participants

All renal transplants performed from January 2001 to December 2010 were included in our retrospective cohort study (n = 762). For the purposes of this study, older people were defined as aging 65 years or older (n = 59) and post-transplantation outcomes in this group were compared to those in younger patients (n = 703).

### Outcomes

The outcome measures studied were: delayed graft function (DGF, defined as the need for dialysis within the first week following transplantation), primary non-function (PNF, as defined by failure of the transplanted kidney to function within the first 6 weeks post-transplant), biopsy proven acute rejection (BPAR), serum creatinine at 1 year and graft and recipient survival. Lengths of initial hospital stay and re-admission rates were also assessed.

#### ***Statistical analysis***

Student’s T-Test was used to analyse continuous variables, Pearson’s Chi-Squared test for categorical variables and the Kaplan-Meier estimator for survival analysis. SPSS (version 16) was used for data analysis.

#### ***Ethics***

Formal ethical approval was not required for this retrospective study as it did not affect the management of its subjects. Approval was obtained from the local Clinical Effectiveness Department to analyse outcomes.

## Results

### Renal transplantation rate and type of donor organ used in older transplant recipients

Older people represented a relatively small proportion of live donor (4.0%) and cadaveric donor (9.1%) renal transplant recipients. 8% of donor organs were from people aged over 65. The proportion of renal transplants where cadaveric donor organs were used became greater as recipient age increased, as shown in Table 1. The total number of renal transplants performed decreased with age despite an increased prevalence of ESRD in older people.

**Table 1:** Number of renal transplants performed by age and proportion of live and cadaveric donor organs used for each age group

**Renal transplant recipient age group**	**Cadaveric donor organ (%)**	**Live donor organ (%)**
Less than 45 years old (n=400)	66.5%	33.5%
45 – 64 years old (n=303)	80.9%	19.1%
65 years old or older (n=59)	86.4%	13.6%

### Demographics and pre-transplant data of older patients undergoing renal transplantation

Of the 59 patients aged 65 years or over undergoing transplantation, the median age was 67 (range: 65 – 75). Thirty seven recipients (62.7%) were male and 22 recipients were female (37.3%). The aetiology of renal failure in the older patient population is shown in Table 2. Forty seven recipients (79.7%) were receiving regular dialysis prior to transplantation for a median duration of 4 years (range: 0.4 – 20 years). In order to be able to adhere to a strict post-operative immunosuppressive drug regimen, all patients listed for transplantation were deemed to have a high level of cognitive function.

**Table 2:** Aetiologies of renal failure in patients aged over 65 years undergoing renal transplantation

**Aetiology of renal failure**	**No. of recipients aged 65 or over (%)**
Unknown aetiology	17 (28.8%)
Glomerulonephritis	12 (20.3%)
Other	8 (13.5%)
Obstructive uropathy	5 (8.5%)
IgA nephropathy	4 (6.8%)
Adult polycystic kidney disease	3 (5.1%)
Hypertensive nephropathy	3 (5.1%)
Diabetic nephropathy	2 (3.4%)
Reflux nephropathy	2 (3.4%)
Chronic pyelonephritis	1 (1.7%)
Focal segmental glomerulosclerosis	1 (1.7%)
Congenital hypoplastic kidneys	1 (1.7%)

#### ***The association between donor organ Age on outcomes following renal transplantation***

The older recipients who did undergo renal transplantation were more likely to receive organs from older donors (27.1% vs. 6.3%; p < 0.001). Kidneys from such donors were more likely to have DGF (40.7% vs. 16.9%; p < 0.001) and fail in the first year after transplantation (15.3% vs. 7.6%; p = 0.04). Serum creatinine at 1 year was also higher in patients who received kidneys from elderly donors (245 + /-29.3 μmoll/l vs. 176.7 + /-6.7 μmol/l; p = 0.02) but there was no association with increased recipient mortality.

#### ***The association between recipient Age and outcomes following renal transplantation***

Older renal transplant recipients were less likely than younger recipients to have BPAR (6.8% vs. 22%; p < 0.01). There was no difference in the rates of DGF (16.9% vs. 18.5%; p = 0.77) or graft loss at 1 year (11.9% vs. 7.8%; p = 0.28) between older and younger recipients. Mortality rate in the first year post transplantation was, however, greater in older patients (6.8% vs. 2.1%; p = 0.03).

The mean length of hospital stay was 7.91 + /-2.49 days. 12.1% of patients were readmitted in the year following renal transplantation. Readmission rates were higher in older renal transplant recipients (31.8% vs. 10.9%; p < 0.001) but not in the recipients of kidneys from older donors (18.6% vs. 11.9%; p = 0.32). Length of initial hospital stay was not affected by donor (7.87 + /- 2.46 days vs. 7.9+/- 2.78 days, p = 0.73) or recipient (7.9+/-2.48 days vs. 8.0+/-2.68 days, p = 0.846) age.

## Discussion

Older people represent a considerable and increasing proportion of adults with ESRD. There is growing evidence that such patients, in the absence of contraindications, have better outcomes after renal transplantation than alternative forms of RRT [2,11]. Previous research highlighted that half of patients receiving RRT in the dialysis units which refer to our transplant unit are aged 65 or over [9]; in this study they represented only 4% and 9.1% of live and cadaveric donor renal transplant recipients respectively.

Outcomes in those elderly patients who did undergo renal transplantation were, however, encouraging. Older transplant recipients were not more likely to have delayed graft function or graft loss in the year following transplantation despite significantly increased usage of older donor kidneys in older recipients. The length of initial post-operative stay after transplantation was not affected by recipient age with the average stay being 7.9+/-2.48 days in patients aged 65 or over and 8.0 + /-2.68 days in patients aged under 65 (p = 0.846). Similarly, Fabrizii et al. [12] reported no significant difference in initial duration of hospital stay between older and younger patients. Whilst this may appear surprising, one of the main reasons for a prolonged hospital stay after renal transplantation is DGF, irrespective of patient age [13]. We have demonstrated comparable DGF rates in older and younger recipients. This may account for the similar durations of hospital stay in the two groups. The rate of readmission was, however, higher than for younger recipients and this may have implications for healthcare planning and service delivery of renal transplantation in the future, with additional hospital beds and resources allocated to post-transplantation care of older renal transplant recipients. Nevertheless the quality of life and economic benefits of transplantation over dialysis (which generally involves frequent hospital attendance [14,15]) are well recognised and will likely result in overall reduction in healthcare costs for these patients. Mortality rate was higher in older transplant recipients, reflecting the expected increased mortality in this group. It is recognised that mortality rate in older people with ESRD is lower when treated with transplantation than alternative forms of RRT [16].

This study also highlights the significantly reduced rate of biopsy-proven acute rejection of renal grafts in older patients. Prevention of rejection is generally easier in older patients due to natural immunosenescence [17], allowing reduced dosage of immunosuppressant medication. Immunosuppressant dose reduction in older renal transplant recipients has been associated with improved recipient and graft survival, reduction in cardiovascular risk, reduced drug side effects and cost savings [18].

Therefore there is increasing evidence that, in the absence of contra-indications, older people with ESRD are best treated with renal transplantation and that some important aspects of post-operative care are less challenging due to natural immunosenescence than for younger patients. Nevertheless, several factors limit increased access to transplantation in older patients. These include organ allocation policies and failure to refer older potential transplant recipients, although the primary factor remains insufficient organ availability. With an average waiting time for a cadaveric kidney in the United Kingdom of around 4 years, there may be a natural reluctance to refer older patients with a shorter life expectancy. Several strategies attempt to address this issue, including using “extended criteria” organs and the Eurotransplant Senior Program, a matching scheme which favours the use of older donor kidneys for older recipients [19]. As our study demonstrates, outcomes from such kidneys are inferior to younger kidneys from healthier donors, with increased DGF, graft failure and serum creatinine levels. This is, however, an acceptable compromise if access to transplantation for older people is improved since most have a functioning graft at time of death [20] and there was no association between the use of older donor organs and mortality in our study.

Another method of increasing access to transplantation involves improving the live donor transplantation rate. Kidneys from older live donors are not inferior to those from younger cadaveric donors [21] and spare the recipient from the morbidity and mortality of dialysis whilst awaiting transplant – this is considerable, with the majority of older people on the transplant waiting list dying before transplantation. Additionally, several studies have found that older people donating kidneys for live donor transplantation experience minimal adverse health effects [22-24]. Older people should therefore not be discouraged from offering kidneys for live donor renal transplantation. Given that only 8% of donors in our study were aged over 65, increased awareness of the excellent reported donor and recipient outcomes following transplantation may increase donation and improve access to transplantation in the future.

The limitations of this study include its retrospective nature, so only the association between age and the outcome measures studied can be calculated and causation cannot be determined. Furthermore, the relatively low transplantation rate in older patients reduces the power of statistical tests to detect small differences in outcomes.

## Conclusions

From our study, we support the fact that fit older people may offer kidneys for live donor kidney transplantation with excellent outcomes for both donor and recipient. We recommend that an increased awareness that older people with ESRD are best treated by renal transplantation in the absence of contraindications is necessary amongst all clinicians responsible for treating older patients.

## Abbreviations

BPAR: Biopsy-proven acute rejection; DGF: Delayed graft function; ESRD: End stage renal disease; PNF: Primary non-function; RRT: Renal replacement therapy.

## Competing interests

The authors declare that they have no conflict of interest.

## Authors’ contributions

NJD: wrote the paper, data analysis, data collection. CDLC: assistance with writing paper, data analysis, data collection. EA: assistance with writing paper, data analysis, data collection. DK: senior author, concept and design of study, final approval for paper to be published. All authors read and approved the final manuscript.

## Pre-publication history

The pre-publication history for this paper can be accessed here:

http://www.biomedcentral.com/1471-2318/13/79/prepub
